# An empirical investigation of deviations from the Beer–Lambert law in optical estimation of lactate

**DOI:** 10.1038/s41598-021-92850-4

**Published:** 2021-07-02

**Authors:** M. Mamouei, K. Budidha, N. Baishya, M. Qassem, P. A. Kyriacou

**Affiliations:** 1 Deep Medicine, Nuffield Department of Women’s and Reproductive Health, Oxford Martin School, John Radcliffe Hospital, University of Oxford, Oxford, OX3 9DU UK; 2 Research Centre for Biomedical Engineering (RCBE), School of Mathematics, Computer Science and Engineering, City, University of London, Northampton Square, London, EC1V 0HB UK

**Keywords:** Biomarkers, Diagnostic markers, Optical physics, Near-infrared spectroscopy, Computational science

## Abstract

The linear relationship between optical absorbance and the concentration of analytes—as postulated by the Beer-Lambert law—is one of the fundamental assumptions that much of the optical spectroscopy literature is explicitly or implicitly based upon. The common use of linear regression models such as principal component regression and partial least squares exemplifies how the linearity assumption is upheld in practical applications. However, the literature also establishes that deviations from the Beer-Lambert law can be expected when (a) the light source is far from monochromatic, (b) the concentrations of analytes are very high and (c) the medium is highly scattering. The lack of a quantitative understanding of when such nonlinearities can become predominant, along with the mainstream use of nonlinear machine learning models in different fields, have given rise to the use of methods such as random forests, support vector regression, and neural networks in spectroscopic applications. This raises the question that, given the small number of samples and the high number of variables in many spectroscopic datasets, are nonlinear effects significant enough to justify the additional model complexity? In the present study, we empirically investigate this question in relation to lactate, an important biomarker. Particularly, to analyze the effects of scattering matrices, three datasets were generated by varying the concentration of lactate in phosphate buffer solution, human serum, and sheep blood. Additionally, the fourth dataset pertained to invivo, transcutaneous spectra obtained from healthy volunteers in an exercise study. Linear and nonlinear models were fitted to each dataset and measures of model performance were compared to attest the assumption of linearity. To isolate the effects of high concentrations, the phosphate buffer solution dataset was augmented with six samples with very high concentrations of lactate between (100–600 mmol/L). Subsequently, three partly overlapping datasets were extracted with lactate concentrations varying between 0–11, 0–20 and 0–600 mmol/L. Similarly, the performance of linear and nonlinear models were compared in each dataset. This analysis did not provide any evidence of substantial nonlinearities due high concentrations. However, the results suggest that nonlinearities may be present in scattering media, justifying the use of complex, nonlinear models.

## Introduction

Near Infared (NIR), Mid-Infrared (mid-IR) visible and Ultraviolet (UV) optical spectroscopy provide a low-cost and non-invasive alternative to electrode-based approaches in the characterization of chemical compounds and the quantification of analytes. Such applications necessitate training predictive models on datasets with sufficient variations in the concentration of absorbing species. However, the provision of such datasets is highly time—and resource-demanding, as a result, the number of samples are often small. Moreover, the identification of discriminative optical patterns requires scanning broad ranges of the optical spectrum. Therefore, the acquired optical spectra contain absorbance values at hundreds or thousands of wavelengths. The limited sample sizes, n, along with the large number of wavelengths (variables), p, constitute the main features of most optical spectroscopy datasets; known as large p small n problems^1^. While this poses a challenge for predictive modelling, two conditions make the problem tractable:

- the presence of redundant variables and the multicollinearity of absorbance values mean the resulting covariance matrices are low rank,
- under uniform attenuation conditions, the Beer-Lambert law postulates a linear relationship between the absorbance of monochromatic light and the concentration of absorbing species,
  1 $$A = \log _{{10}} I_{0} /I = l\sum\limits_{{i = 1}}^{N} {\epsilon_{{\text{i}}} } c_{i} ,$$

where A denotes absorbance, $I_{o}$ and $I$ are the intensity of beam before and after passing through the absorbing layer, $N$ is the number of absorbing species in the matrix, $\epsilon _{i}$ is the molar decadic extinction coefficient for the i^th^ absorbing species, $c_{i}$ is the concentration, and $l$ is the path length of light.

These considerations justify the choice of Principal Component Regression (PCR) and Partial Least Squares (PLS) in spectroscopic studies^2,3^. Both of these methods are linear. The former achieves dimensionality reduction by finding the axes of maximal variance in the space of independent variables, $X_{{n \times p}}$, while the latter does so by finding the axes of maximal covariance between the independent variables and the dependent variable, $Y_{{n \times 1}}$. In spite of major differences in the interpretation of latent variables in PCR and PLS, they often deliver similar predictive performances^4^. Minor improvements in predictive performance might be expected from PLS, particularly when noise constitutes much of the variance in the independent variables space^5–7^.

While PLS and PCR remain workhorses of quantitative analytics in spectroscopic studies, it is also well-understood that deviations from the linearity assumption can take place when the light source is not monochromatic, the concentration of the analytes are high, and the medium is highly scattering. Mayerhöfer et al.^8^ showed errors that arise from the Beer-Lambert law can exceed an order of magnitude compared to the exact solution of the Maxwell equations. Tolbin et al.^9^ derived analytical expressions for the critical concentration and the extinction coefficient beyond which deviations from the Beer-Lambert law become significant. Firstly, the expectation of nonlinearities that are challenging to quantify apriori, and secondly, the prevalence and success of nonlinear Machine Learning (ML) models in different fields have paved the way for their application in spectroscopic studies. For instance^10^, used Artificial Neural Networks (ANN) for classification of drug strength from NIR spectra. Santana et al.^11^ compared the application of discriminant PLS and Random Forest (RF) on classification of adulterated oil and spice samples from Fourier Transform Infrared (FT-IR) and NIR spectra. They reported that RF delivers a superior performance. Mekonnen et al.^12^ compared the performance of methods such as PLS, Support Vector Regression (SVR), ensemble decision trees, and ANNs on the estimation of the concentration of glucose in aqueous solutions. This comparison was conducted on an NIR dataset comprising of 47 concentrations of glucose. SVR, ANN, and a variant of ensemble decision trees obtained better performances. Balabin and Lomakinab^13^ compared the performance of PLS, SVR, and ANN on NIR spectra obtained from different petrochemical matrices. It was shown that ANN and SVR deliver comparable performances and both offer more accurate predictions than PLS; the authors concluded that SVR can provide a robust alternative to ANN. Similar investigations of linear and nonlinear regression models have been reported in the literature for the estimation of soil carbon content, sugar content of orange, active substance content of tablets, moisture, fat and protein content of meat, and finally protein content of wheat in^14–16^. The PLS model is often found to deliver poorer performance compared to nonlinear models.

The present study focuses on lactate. The association of lactate with one of the most fundamental processes in the body, namely cellular respiration, makes it an important biomarker akin to glucose. Therefore, not surprisingly, clinical literature underlines the diagnostic and prognostic value of lactate in relation to numerous life-threatening conditions and diseases, such as sepsis, diabetes, cancer, pulmonary and kidney diseases^17–20^. Lactate has also been referred to as an important indicator of the risk of morbidity and mortality in critically ill patients^21^. Currently, the gold standard in the measurement of lactate requires blood sampling. This limits the ability of intensivists to frequently monitor patients’ lactate levels; in spite of the calls for its routine measurements in patients with sepsis^22^. These considerations have given rise to the pursuit of non-invasive and continuous alternatives to intermittent blood sampling for lactate measurement.

Petibois et al.^23^ used the mid-IR region of the optical spectrum to estimate the concentration of lactate in plasma and reported a coefficient of determination, $R_{T}^{2}$, of 0.94 in the test set and a Root Mean Square Error of Prediction (RMSEP) of 0.15 mmol/L. Lafrance et al.^24^ showed the potential of NIR spectra in the estimation of lactate concentration in blood, reporting a coefficient of determination of 0.96 with cross-validation,$R_{{CV}}^{2}$. Mamouei et al.^25^ applied a number of variable selection methods to the mid-IR spectra of lactate and showed that highly accurate estimates, $R_{{CV}}^{2} = 0.996$, can be achieved with models that only use a small subset of wavelengths. Budidha et al.^26^ conducted a comprehensive comparison of the different regions of the optical spectrum, namely ultraviolet/visible, NIR, and mid-IR, for the measurement of lactate and highlighted the merits of mid-IR for in-vitro applications and NIR for transcutaneous applications.

In this study we adopt an empirical approach to investigate potential deviations from the Beer-Lambert law that arise from high concentrations of lactate and scattering matrices. To this end, we compare the performance of linear models, namely PCR, PLS, and linear SVR, with nonlinear models, specifically, SVR with quadratic, cubic, quartic, and Radial Basis Function (RBS) kernels. To isolate the effects of high concentrations, this comparison is performed on three partially overlapping datasets comprising of NIR spectra of lactate in PBS with concentrations in the range of 0–10 mmol/L, 0–20 mmol/L and 0–600 mmol/L. To investigate the effects of scattering matrices, the comparison is extended to incrementally more scattering matrices; from PBS to serum, whole blood, and in vivo transcutaneous spectra.

## Results

The performance of seven linear and nonlinear models are compared to investigate the extent of nonlinearities caused by high concentrations and scattering matrices. The hypothesis is that if these factors introduce significant nonlinearities, nonlinear models are expected to perform better compared to linear models.

The performance of models is evaluated using cross-validation, with test sets of size three in each iteration. These test sets are randomly selected with uniform distribution and without replacement. This is referred to as the model evaluation cross-validation loop. For SVR models, hyperparameter optimization (to find the values of C, $\epsilon$ , and the kernel scale) is performed within each fold with another five-fold cross-validation routine and Bayesian optimizer. This (hyperparameter optimization) cross-validation is nested inside the model evaluation cross-validation loop to ensure that the prediction results are representative of the external predictive performance while minimizing the risk of hyperparameter misspecification. Firstly, the model evaluation cross-validation ensures that the predictive performance of each model is tested across all samples. The alternative approach of using a single test set, given the small sample size, can be susceptible to outliers and the selection of unrepresentative tests sets, therefore, it can lead to unreliable measures of predictive performance. Secondly, the nested hyperparameter tuning cross-validation reduces the risk of hyperparameter misspecification. The alternative approach of using a randomly selected validation set would result in less samples in the training set. Also, similarly, a single validation set might not be representative of the spectra and, therefore, lead to hyperparameter misspecification. After the completion of the model evaluation cross-validation loop, Root Mean Square Error of Cross-Validation (RMSECV) and $R_{{CV}}^{2}$ are calculated. Note the $R_{{CV}}^{2}$ presented here is the coefficient of determination pertaining to the cross-validation routine and is different from the $R^{2}$ of calibration. This assesses the goodness of fit between the predicted values of the holdout spectra in the cross-validation routine and their reference values. Since in the model evaluation cross-validation routine, each spectrum is held out and predicted exactly once, the $R_{{CV}}^{2}$ demonstrates the predictive performance across all spectra.

As a result of the random sampling of the training and test sets within both cross-validation loops and due to the stochastic nature of the Bayesian optimizer, different hyperparameters and models may be found in different runs, leading to different results (RMSECV and ${R}_{CV}^{2}$). Therefore, the process (i.e. the main model evaluation cross-validation) is repeated 10 times; ${R}_{CV}^{2}$ values are visualized with boxplots and the lowest RMSECV among all ten runs is reported separately in Table 1. The presentation of results as boxplots helps capture the stochastic nature of the results as well as the convergence properties of the optimization in SVR models; a wider spread implies poorer convergence and model misspecification. Capturing this aspect is particularly important, as it is a direct cost of using more complex models compared to PLS and PCR. The inclusion of these considerations in the present study distinguishes the results presented here from our previous preliminary work on the PBS spectra^27^.

**Table 1:** The comparison of pls, pcr and svr models with diefferent kernel functions in different matrices.

Model	RMSECV [mmol/L]					
	PBS			Human Serum	Sheep Blood	Invivo
	0–11 mmol/L	0–20 mmol/L	0–600 mmol/L	7.7–15 mmol/L	4.8–13.8 mmol/L	1.1–11.7 mmol/L
PLS	0.88	0.87	1.61	1.11	1.58	1.03
PCR	1.61	0.88	1.65	1.35	1.66	1.12
SVR-linear	**1.60**	**0.87**	**1.53**	1.23	1.65	**1.08**
SVR-quadratic	1.95	1.21	12.87	1.22	1.70	1.21
SVR-cubic	1.68	1.37	15.20	**1.15**	1.60	1.37
SVR-quartic	1.86	1.61	8.53	1.28	1.66	1.42
SVR-RBF	1.77	2.18	38.25	1.32	**1.54**	1.30

In the interpretation of the results, it is worth noting that the PLS model is fitted directly on the spectra while the PCR and all SVR models are fitted to the PCs that present a loss of 0.01% in variance in the PBS, serum, and blood datasets, and a loss of 20% in the invivo dataset. Therefore, a major difference between the performance of PLS and PCR models can imply that a) the variance not captured by the PCs are important for predictions b) the ratio between the number of PCs and the number of observations is large, leading to poorer regression. Hence, the assessment of linear and nonlinear effects should be primarily based on the comparison of how well the PCR and the SVR with linear kernel perform relative to the nonlinear models. A fairer comparison may take the hyperparameter tuning requirements of the SVR models into account, restricting pairwise comparisons to SVR with linear kernel to SVR with nonlinear kernels.

### Nonlinearities due to high concentration

**Figure 1:**
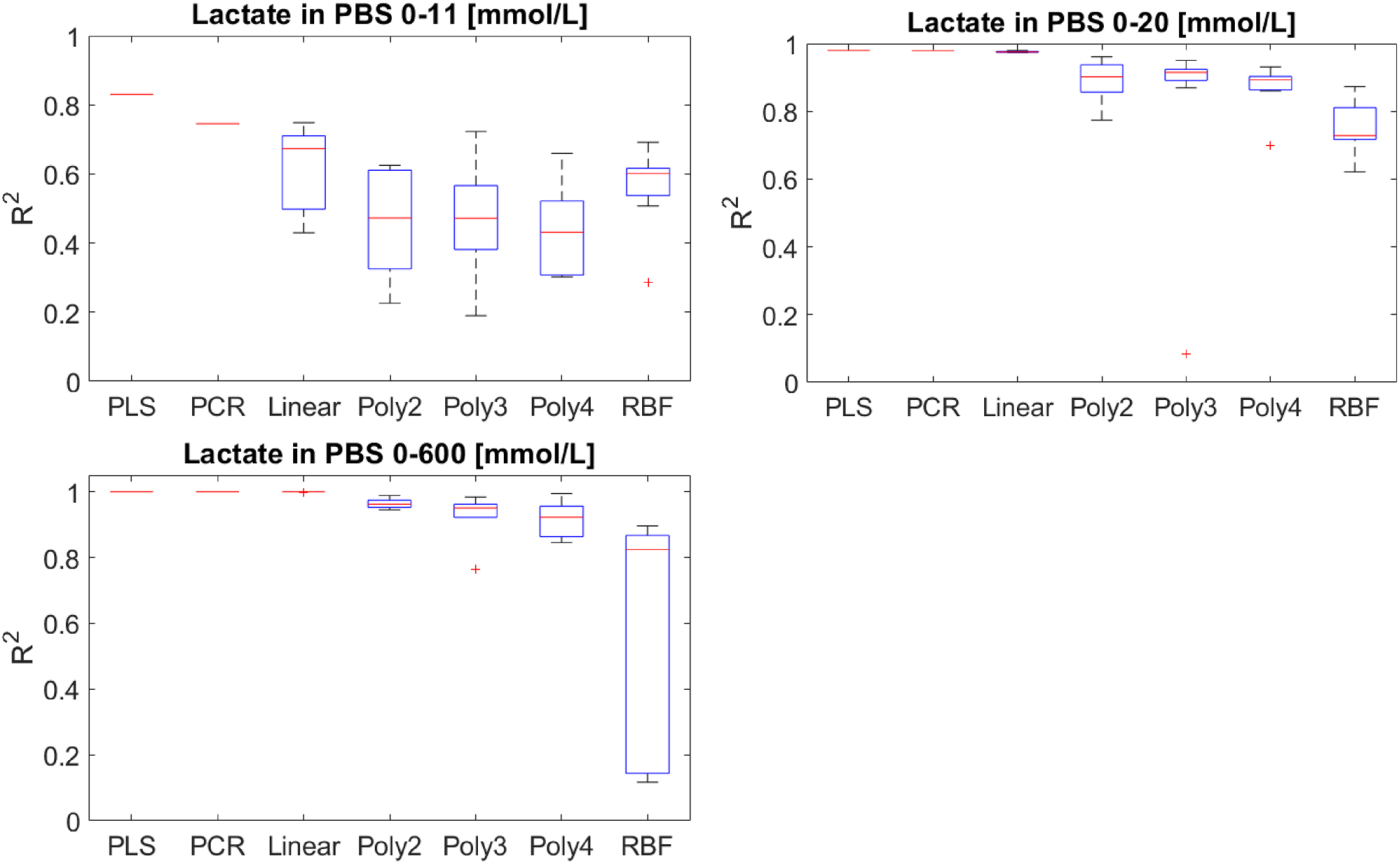
The comparison of the performance of linear and nonlinear models is datasets with low, medium, and high ranges of lactate concentrations. The boxplots summarise the results pertaining to ten runs of cross-validation for each model. After each run, an estimate of the coefficient of determination, ${R}_{CV}^{2}$, is obtained.

To investigate the effect of high concentrations of lactate, first the PBS dataset is analyzed where scattering due to compounds other than lactate is minimal. To this end, three partially overlapping datasets with concentrations of lactate ranging between 0–11, 0–20, and 0–600 mmol/L are used to compare the performance of linear and nonlinear regression methods. The choice of the ranges of lactate levels was primarily motivated by computational considerations. The approach described above for hyperparameter tuning and model evaluation, while delivers more reliable results, is very computationally demanding. Performing this for SVR models takes multiple days for each dataset on a high-end workstation with a 10-core Intel® Xeon® Silver 4114 processor and 32 GB RAM . The preferable approach would be to incrementally increase the range of the dataset—by adding one or multiple samples at the time—and analyzing the trends in model performance, however, due to the high computational time this was not feasible. Instead, we allocated the spectra to the three aforementioned sets to present significant increase in the range of lactate levels. Since the model evaluation cross-validation loop sampled test sets of threes, minor adjustments were made to keep the number of spectra in the sets divisible by three. The third dataset was formed by adding six spectra pertaining to lactate concentrations of 100, 200, 300, …, and 600 mmol/L to the data. These values are far outside the physiological range of lactate, but they were included to assess if extremely high concentrations of lactate cause nonlinearities. Figure 1. Depicts the results.

In all datasets—specifically in the datasets with medium and high concentrations of lactate—the linear models obtain the best performances. Therefore, this analysis does not provide any evidence of the presence of significant nonlinearities due to high concentrations of lactate.

### Nonlinearities due to scattering matrices

**Figure 2:**
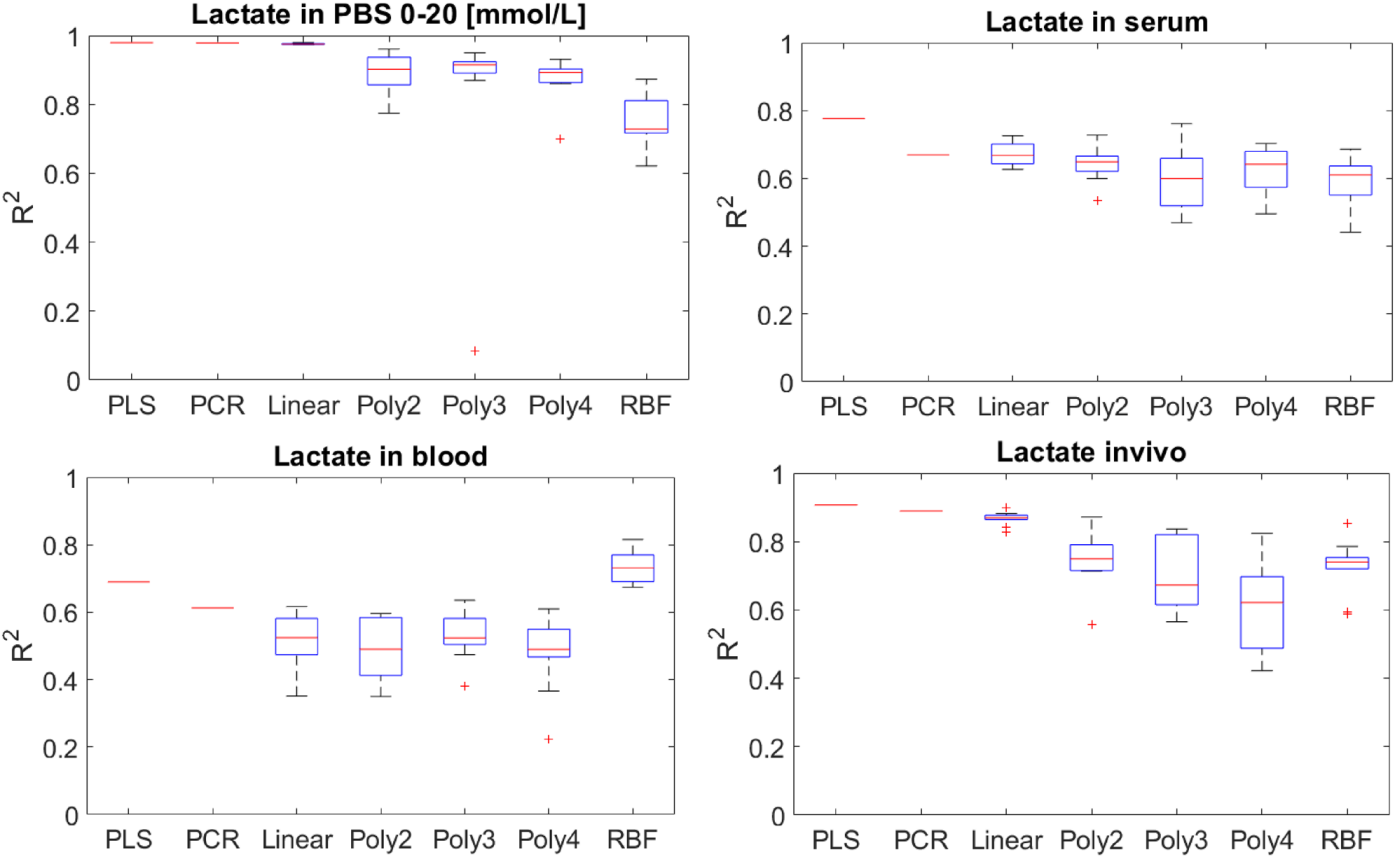
The comparison of the performance of linear and nonlinear models in increasingly more scattering matrices. The boxplots summarise the results pertaining to ten runs of cross-validation for each model. After each run, an estimate of the coefficient of determination, ${R}_{CV}^{2}$, is obtained.

Figure 2, summarizes the performance of models in increasingly more scattering matrices, namely PBS, human serum, sheep blood and in transcutaneous spectra acquired from the participants of an exercise study.

While the results do not show a strong upward trend with increasing model nonlinearity, both in the serum dataset and the blood dataset interesting patterns emerge. Unlike PBS, in serum and blood, whether the best observed $R_{{CV}}^{2}$ in ten runs (top end of the top whisker) or the median $R_{{CV}}^{2}$ (the red bar) are considered, the trend is either almost flat or upward. In serum, the SVR model with cubic polynomial kernel shows a better performance than the linear SVR and PCR models when the top whisker is considered. This effect is much more pronounced in the blood dataset. The SVR model with RBF kernel obtains the best $R_{{CV}}^{2}$, in terms of both the median and the best observed case. This is despite the fact that in small datasets, the complexity and the requirement of hyperparameter tuning for the SVR models puts them at a disadvantage compared to simpler PCR and PLS. This suggests that the nonlinearities must be substantial to compensate for and exceed the expected marginal loss of accuracy due to additional model complexity. The noticeably high nonlinearity observed in sheep blood relative to human serum, could be related to the differences between the two mediums. The composition of blood and the distribution of lactate between red blood cells and plasma are different in the two mediums, potentially contributing to differential scattering properties and optical absorbance profiles.

Surprisingly, the same pattern does not emerge in the invivo dataset. One possible explanation is that proxies of lactate concentration might have been detected rather than lactate itself. In the exercise study it was expected that other absorbing species, such as oxyhaemoglobin and deoxyhaemoglobin, can show highly correlated variations with lactate. The observations that RMSECV is lower and $R_{{CV}}^{2}$ is higher in this dataset relative to serum and blood, supports this possibility. Especially because in this dataset, a portable spectrophotometer with a much lower resolution and a shorter wavelength range was used, the number of observations was smaller, and baseline differences in optical properties of the four participants are expected to be far greater. Table 1. Shows the best RMSECV obtained in ten different runs of the cross-validations routine.

## Discussion and conclusion

This study focused on the optical behavior of lactate; an important and fundamental biomarker that, if measured routinely and accurately, can shed light on numerous diseases and health problems. Previous studies have underlined the potential of optical estimation of lactate as a noninvasive, inexpensive and continuous alternative to blood sampling. However, accurate and reliable optical measurements are still not within reach. A better understanding of the interactions of light and this biomarker is necessary to recognize the merits, limitations, and practical issues of optical sensing. Since previous attempts in the optical estimation of lactate have used linear models, we investigated potential deviations from the linearity assumption postulated by the Beer-Lambert law. To this end, a series of experiments were conducted to analyze potential nonlinear effects that can arise due to high concentrations of lactate and scattering matrices. The assumption was that if nonlinear effects become substantial, nonlinear models will deliver better accuracies than linear models.

Seven linear and nonlinear models were compared in datasets with concentrations of lactate ranging between 0–11 mmol/L, 0–20 mmol/L, 0–600 mmol/L. For this investigation a minimally scattering matrix (PBS) was used to ensure potential deviations are mainly due to high concentrations. This analysis did not provide any evidence of significant nonlinearities. A similar comparison in incrementally more scattering matrices, namely human serum, sheep blood, and transcutaneous spectra, showed some merits to the use of nonlinear models. Both in serum and blood, nonlinear models obtained better performances than PCR and SVR with linear kernel.

In summary, (a) the results confirm the potential of optical measurements of lactate, both invitro and invivo, albeit the latter is likely to have been indirect. Therefore, more studies, with more participants, and in different scenarios is necessary to assess the feasibility of indirect optical sensing of lactate. (b) It was shown that concentrations of lactate, even far beyond the biological range, do not present substantially nonlinear absorbance. (c) Nonlinear models showed merits in direct measurement of lactate when the medium is scattering, i.e. serum and blood.

Finally, the authors would like to emphasize that the RMSECV presented in Table 1 is not representative of the best accuracies that can be achieved. Previous studies have shown that major improvements may be observed when nonlinear baseline corrections are used and redundant wavelengths are excluded. In the present study, since the primary objective was the analysis of nonlinear absorbance, the aforementioned topics were not covered.

## Methods

### The datasets

All datasets were produced after obtaining approval by the Senate Research Ethics Committee (SREC) at City, University of London (SREC 17–18 05 6ii 27.06.2018) and all methods were carried out in accordance with the relevant guidelines and regulations.

#### PBS

The dataset consists of 57 NIR spectra of different concentrations of lactate in a Phosphate Buffer Solution (PBS). The procedure for the preparation of the solutions and acquisition of the spectra is detailed in^27^. The dataset contains 31 samples with concentrations of lactate between 0–10 mmol/L (increments of 0.25 mmol/L), 21 samples between 10.5–20 mmol/L (increments of 0.5 mmol/L) and finally, six samples with extremely high concentrations of 100–600 mmol/L (increments of 100 mmol/L). Spectra were acquired using the Lambda 1050 dual-beam UV/Vis/NIR spectrophotometer (Perkin Elmer Corp, Massachusetts, USA), with a spectral resolution of 1 nm. The light source used in the spectrophotometer was a halogen tungsten lamp. Indium gallium arsenide (InGaAs) and the lead sulfide detectors (PbS) were used to detect the transmitted NIR light in the range between 800 and 2600 nm. Baseline correction was performed on the spectrophotometer prior to the acquisition of a spectra, at 100% Transmission / 0% absorbance to remove background noise. Once, background correction was performed, 300 µl of each sample was transferred to in a macro quartz cuvette (Hellma GmbH & Co.KG, Jena, Germany) with a path length of 1 mm to acquire the three NIR spectra of each sample in the desired wavelength range. The three spectra were then averaged, and the resulting spectrum of each sample was considered for further analysis.

#### Serum

The dataset consists of 36 NIR spectra of lactate in human blood serum. Mixed pool human serum collected from healthy volunteers was purchased from TCS Biosciences Ltd., (Buckingham, UK). The base lactate of the purchased serum was 7.7 mmol/L. Thirty-five serum samples of 29 mL were then serially diluted with 1 mL of stock solutions containing varying concentration of lactate in PBS. The concentration of the serum samples was measured before the acquisition of spectra using the ABL 825 Flex (Radiometer UK Limited, Crawley, West Sussex, UK). The concentration of lactate within the samples ranged between 7.7 and 15 mmol/L with an average increment of 0.20 mmol/L.

Once the samples were prepared and the concentration measured, the spectra of each sample was acquired again in transmission mode using the Lambda 1050 dual-beam UV/Vis/NIR spectrophotometer (Perkin Elmer Corp, Massachusetts, USA). The acquisition procedure of serum spectra was similar to that of the PBS spectra, as detailed above.

#### Sheep blood

The dataset consists of 36 spectra of lactate in sheep blood acquired using the Lambda 1050 dual-beam UV/Vis/NIR spectrophotometer (Perkin Elmer Corp, Massachusetts, USA) equipped with 100 mm InGaAs Integrating Sphere. All the spectra were acquired in reflectance mode using a 300 µl sample in the range between 900 and 2500 nm. The procedure for the preparation of the solutions and acquisition of the spectra is detailed in^28^. The concentration of lactate is within the range of 4.8–13.8 mmol/L and the average increment is around 0.25 mmol/L. The spectral resolution and acquisition setup used to acquire blood spectra were similar to that of PBS.

#### Invivo

The dataset consists of 27 reflectance spectra obtained from four healthy participants during maximal effort cycling on a spinning bike (WattBike Pro, Wattbike Ltd, Nottingham, UK). The participants were 22–31 years old and gave informed written consents before commencing the experiment. Since blood lactate is known to increase with physical effort, the experiment was designed to induce changes in participants’ blood lactate levels over the course of the exercise study. The participants were asked to cycle for as long as they can at a fixed peddle speed and magnetic resistance, the air resistance was increased after every minute until volitional exhaustion of the volunteer or upon reaching 90% of predicted maximal heart rate (derived using the equation max heartrate = 220 − age in years). A 45 s rest was allowed after every minute of cycling. During the rest period, optical spectra were acquired from the right index of the volunteer and a drop of blood was drawn from the left index finger using a sterile lancet. The capillary blood was collected on a finger stick and the lactate level in blood was measured using the portable Lactate Pro 2 analyzer. The concentration of lactate measured was within the range of 1.1–11.7 mmol/L and the average increment was 0.41 mmol/L. As intended, overall, the participants’ lactate levels followed an upward trend, however, significant variations in patterns of increase and maximum lactate levels were observed amongst the participants. For instance, for one of the participants, who is a semi-professional cyclist, the highest lactate level was around 4 mmol/L after 20 min of exercise, while another volunteer reached exhaustion and a lactate of around 8 mmol/L only after 5 min.

Invivo spectra in the 900–1700 nm range were acquired using the NIRQUEST 512–1.9 NIR Spectrophotometer (Ocean Optics Inc., Florida, USA). A reflectance optical fiber probe (600 um fibers) was used to transmit and detect NIR light reflected from the finger of health volunteers. A small slit of 25 µm was chosen to improve optical resolution and stop the detector from saturating. While it would be preferable to use the same spectrophotometer across all datasets, NIRQUEST was selected for the invivo study due to its high sampling rate. This allowed us to collect a sufficiently large number of spectra during the 45-s rest periods and average them to minimize the motion artifacts. The same could not be achieved with the Lambda 1050 spectrophotometer that has a much lower sampling rate.

**Figure 3:**
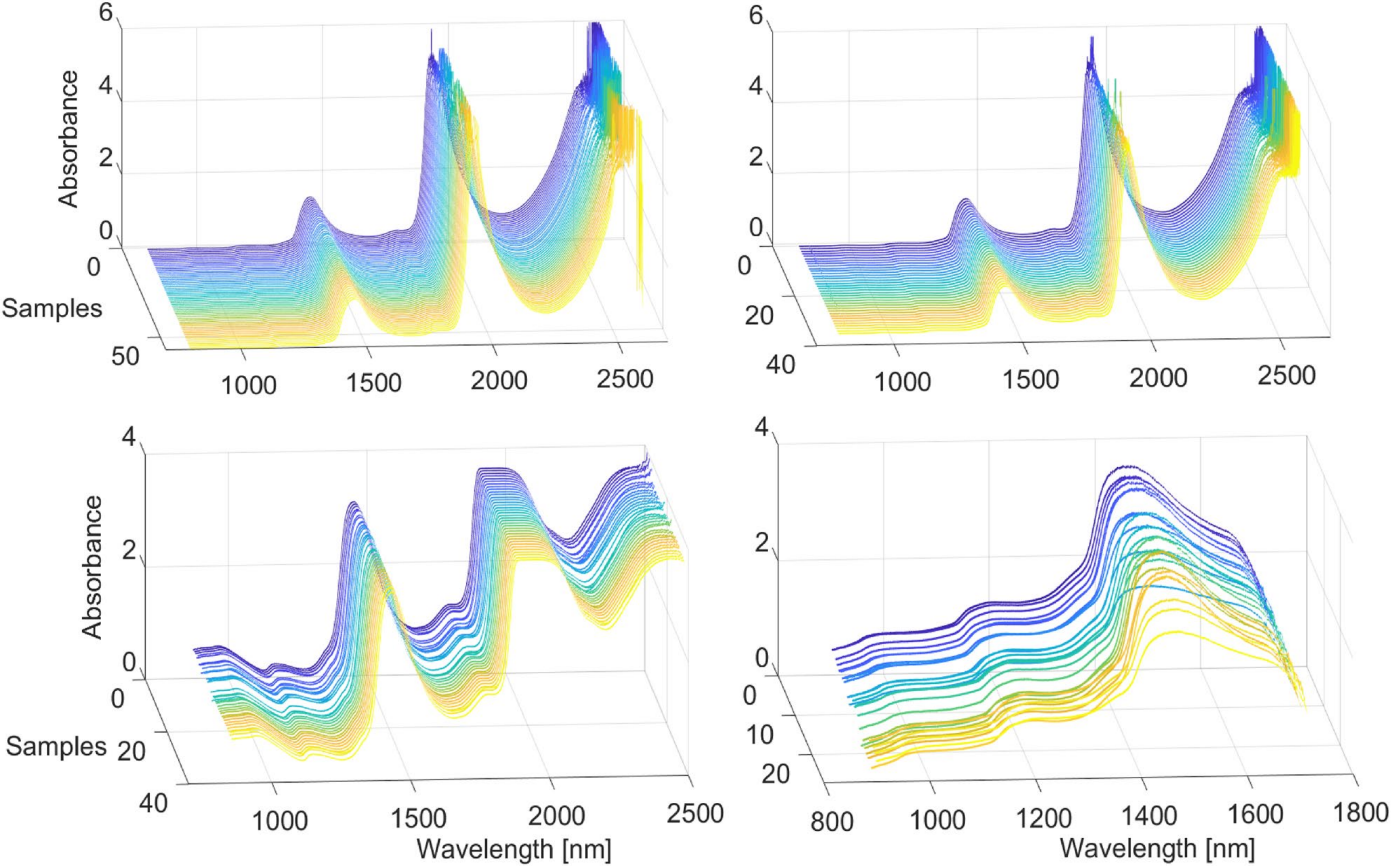
Raw absorbance of spectra of lactate in (**a**) PBS (**b**) human serum (**c**) sheep blood (**d**) invivo human tissue.

Figure 3. depicts the raw spectra in each dataset.

### Preprocessing of spectra

In the PBS and serum datasets, wavelengths between 1900–1980 nm and 2450–2600 nm show high levels of noise and are hence removed. This noise is caused by the oversaturation of the lead sulfide detector in the transmittance mode due to water absorption peaks. The PBS spectra were processed using Multiplicative Scattering Correction (MSC) and a Savitzky-Golay (SG) filter with the window length of 135, second order polynomial and second order derivative. The serum spectra, were processed with MSC and SG filter with window length of 151, third order polynomial and second order derivative. The blood spectra were acquired in reflectance mode and subsequently transformed to absorbance. Therefore, no noise was observed in the aforementioned regions, however, for consistency, they were removed. The transcutaneous spectra were processed with SG filter, polynomial order of three, window length of seven and derivative order of three.

### Dimensionality reduction

Principal Component Analysis (PCA) is applied to all datasets to reduce the dimensionality of the spectra prior to model fitting. For the in-vitro sets the number of components is selected such that 99.99% of the variance is explained by the PCs. This led to the selection of 12, 14, 13 PCs in the PBS datasets with low, medium, and high ranges of concentrations respectively, 16 PCs in the serum dataset, and 22 PCs in the blood dataset. For the tissue dataset, given the noisy nature of the data, this criterion led to a large number of PCs and, consequently, overfitting. The explained variance of 80% was found to produce good results across all models and was therefore selected. This led to 8 PCs.

For the PLS model the number of Latent Variables (LVs) was selected as the point where the Predicted Residual Error Sum of Squares (PRESS) plateaus. This criterion led to 11, 9 and 8 LVs for the PBS datasets with low, medium, and high ranges of concentrations respectively, 10 LVs for the serum dataset, 12 LVs for the blood dataset, and 6 LVs for the invivo dataset.

### Linear and nonlinear models

The linear models used in this study are of PCR, PLS, and SVR,

2 $${f}( {x} ) = {w}\cdot{x} + {b},$$

In SVR the objective is to find the flattest line while the prediction error shows minimal deviation beyond $\epsilon$. Therefore, given the training set $\left( {x_{i} ,y_{i} } \right)$, $i \in \left\{ {1,2, \ldots ,n} \right\}$, $w^{*}$ is defined as

3 $$\begin{aligned} w^{*} & = \mathop {\min }\limits_{{\arg w}} \left\{ {\frac{1}{2}w^{2} + C\mathop \sum \limits_{{i = 1}}^{n} (\zeta _{i} + \zeta _{i}^{*} )} \right\} \\ & \quad {\text{s}}{\text{.t}}{\text{.}}\left\{ {\begin{array}{*{20}c} {y_{i} - w.x_{i} - b \le + \zeta _{i} } \\ {w.x_{i} + b - y_{i} \le + \zeta _{i}^{*} } \\ {\zeta _{i} ,\zeta _{i}^{*} \ge 0} \\ \end{array} } \right. \\ \end{aligned}$$

where $\left\| {\; \cdot \;} \right\|$ is the Euclidean norm, and $\zeta _{i}$, $\zeta _{i}^{{\text{*}}}$ are slack variables that absorb excess errors when a solution is not possible that guarantees errors restricted to $\epsilon$ boundaries. With an $\epsilon$-insensetive loss, $\zeta _{i}$ is defined as,

4 $$\zeta _{i} = \left\{ {\begin{array}{*{20}l} 0 \hfill & {if\;\left| {y - f\left( x \right)} \right| \le \epsilon } \hfill \\ {\left| {y - f\left( x \right)} \right| - \epsilon } \hfill & {otherwise} \hfill \\ \end{array} } \right.,$$

where C is the capacity control parameter that determines the tradeoff between higher loss values and higher norm $w^{2}$ (less flat plane).

The incorporation of nonlinearities can be achieved by using nonlinear transformations, $\phi \left( x \right)$, to map the explanatory variables into new hyperdimensinoal spaces. For instance, a quadratic polynomial augmentation of a two-dimensional feature space ($x_{1}$,$x_{2}$) $\subset {\mathbb{R}}^{2}$ may include all polynomial terms of degree two ($x_{1}$,$x_{2} ,x_{1}$.$x_{2}$, $x_{1}^{2}$,$x_{2}^{2}$) $\subset {\mathbb{R}}^{5}$. In high-dimensional data the explicit use of such nonlinear transformations, $\phi \left( x \right),$ can be intractable. However the use of the “kernel trick” provides a computational effective way to achieve this. Specifically, solving the optimization above necessitates the calculation of $\phi \left( x \right)^{T} .\phi \left( {x'} \right)$, these computationally demanding transformations can be avoided by finding the equivalent kernel, $K\left( {x,x'} \right)$ = $\phi \left( x \right) \cdot \phi \left( {x'} \right)$. Therefore, the four main parameters that need to be selected are the scaling of features, the kernel function, $K\left( {x,x'} \right)$, the loss function, $\epsilon$ , and the capacity control parameter, $C$^29,30^.

In the present sudy, different kernel functions are compared, namely polynomial kernels,

5 $$K_{{poly}} \left( {x,x^{\prime}} \right) = \left( {1 + x^{T} .x^{\prime}} \right)^{p} \;\;\;\;p \in \left\{ {2,3,4} \right\},$$

and Radial Basis Function (RBF),

6 $$K_{{RBF}} \left( {x,x^{\prime}} \right) = e^{{ - \left( {x - x^{\prime}} \right)^{T} \cdot \left( {x - x^{\prime}} \right)}} .$$

The value of C, $\epsilon$, and the kernel scale are optimized.
